# Recent Advances in Understanding the Mechanisms of Therapy-Related Cardiovascular Events in Pediatric Hematologic Malignancies

**DOI:** 10.31083/RCM47353

**Published:** 2026-04-09

**Authors:** Wujian He, Jianhua Feng, Ronghua Luo, Fengchun Jiang, Xiangqian Sui, Xulin Hong

**Affiliations:** ^1^Department of Cardiology, Hangzhou Red Cross Hospital, 310003 Hangzhou, Zhejiang, China; ^2^Department of Pediatric Hematology and Oncology, Women and Children's Hospital Affiliated to Ningbo University, 315012 Ningbo, Zhejiang, China; ^3^Department of Cardiology, Sir Run Run Shaw Hospital, School of Medicine, Zhejiang University, 310000 Hangzhou, Zhejiang, China

**Keywords:** pediatric hematologic malignancies, cardiotoxicity, mechanisms, therapeutic targets, cardioprotective strategies

## Abstract

This narrative review synthesizes the most recent advances in elucidating the mechanisms underlying cardiovascular events associated with the treatment of pediatric hematologic malignancies. First, this review delineates the principal therapeutic modalities currently employed, including chemotherapy, radiotherapy, cellular immunotherapy, and small-molecule targeted therapy. Subsequently, this review offers a systematic and nuanced appraisal of the mechanisms through which these treatments precipitate cardiovascular injury, encompassing direct cardiotoxic effects, inflammatory activation, and immune-mediated tissue damage. Finally, this review examines emerging therapeutic targets with potential relevance for intervention, to refine treatment strategies, mitigate cardiovascular adverse effects, and enhance both quality of life and long-term outcomes in affected children. This review integrates these mechanisms within a cohesive, pediatric-specific conceptual framework and highlights actionable cardioprotective targets across therapeutic modalities.

## 1. Introduction

Pediatric malignancies present marked clinical management challenges due to their particular age of onset and the complexity of their treatment. International registry data from IICC-3 (2001–2010) show that, among children aged 0–14 years worldwide, leukaemias are the most frequent malignancy (world standardised incidence rate 46.4 per million person-years), followed by central nervous system (CNS) tumours and lymphomas (15.2 per million out of an overall rate of 140.6 per million); together, leukaemias and lymphomas therefore account for just over two-fifths of all registered childhood cancers [1], Consistent with this pattern, acute lymphoblastic leukaemia (ALL) is the single most common pediatric cancer globally, representing about 25% of all cancer diagnoses in children younger than 15 years [2]. Advances in multidisciplinary treatment modalities, including surgery, radiation therapy (RT), cytotoxic drugs (such as anthracyclines, alkylating agents, antimetabolites, and plant alkaloids), cellular immunotherapy, and small-molecule targeted therapy, have improved survival rates in pediatric patients [3]. However, it can also lead to cancer therapy-related cardiovascular toxicity (CTR-CVT). Clinical manifestations of CTR-CVT include cardiac dysfunction, myocarditis, abnormal blood pressure, and arrhythmias [4, 5]. Beyond individual case reports, CTR-CVT affects a clinically meaningful proportion of children receiving anticancer therapy and has become a major driver of non-cancer morbidity and late survivorship burden. Population-based survivorship data-predominantly derived from mixed pediatric-adolescent cohorts-indicate that cardiovascular late effects remain highly prevalent even decades after treatment and contribute to excess chronic health conditions in childhood cancer survivors [4]. In the treatment phase, in pediatric treatment protocols, cardiotoxicity may necessitate dose modification or temporary interruption of therapy, potentially compromising oncological efficacy. This may lead to further complications, including heart failure, arrhythmias, or vascular problems. Contemporary cardio-oncology guidance, therefore, emphasises the importance of early recognition and structured surveillance to minimise acute decompensation while preserving oncological efficacy [5].

In the course of the management of pediatric hematologic malignancies, exposure to cytotoxic agents (e.g., anthracyclines and alkylating agents), chimeric antigen receptor T-cell (CAR-T) therapy, immune checkpoint inhibitors (ICIs), and RT has been demonstrated to induce both acute and late cardiotoxicity. The manifestations of this phenomenon include acute myocardial injury, myocarditis, thromboembolic events, cardiomyopathy, heart failure, and cardiac arrhythmias [6, 7]. Notwithstanding the noteworthy advancements in mechanistic research in recent years, the molecular and cellular underpinnings of cardiovascular injury in the pediatric population remain incompletely elucidated, and effective clinical interventions are still lacking.

This review will methodically synthesize the pathophysiological mechanisms of cardiovascular injury attributable to the major therapeutic modalities used for pediatric hematologic malignancies. These modalities include cytotoxic chemotherapy, RT, CAR-T, allogeneic hematopoietic stem-cell transplantation (Allo-HSCT), ICIs, and small-molecule targeted agents. By drawing on recent advances, the review will highlight candidate therapeutic targets to inform risk stratification, surveillance, and the long-term management of cardiovascular health in children.

This review is presented as a narrative synthesis. A structured literature search was conducted in PubMed using combinations of the terms “pediatric”, “hematological malignancies”, “cardiotoxicity”, “anthracyclines”, “radiotherapy”, “CAR-T”, “immune checkpoint inhibitors”, “HSCT”, and “targeted therapy”. Searches were limited to studies published between 2000 and 2024. Both pediatric-specific clinical studies and mechanistic preclinical evidence relevant to pediatric treatment exposures were included. Formal systematic-review methods (e.g., Preferred Reporting Items for Systematic Reviews and Meta-Analyses [PRISMA]) were not applied, as the objective was to provide an integrative mechanistic overview rather than an exhaustive evidence synthesis.

## 2. Therapeutic Modalities and Cardiotoxic Mechanisms in Pediatric Hematologic Malignancies

### 2.1 Cytotoxic Drugs

#### 2.1.1 Overview of Cardiotoxic Cytotoxic Agents

Cytotoxic backbones remain integral to pediatric leukaemia/lymphoma protocols. It is evident that anthracyclines, alkylating agents, antimetabolites and plant alkaloids carry distinct cardiovascular risk signatures that are highly relevant to pediatric practice. The ensuing discourse will be oriented by a concise summary of the representative agents, predominant clinical phenotypes and canonical mechanisms, as delineated in Table 1 [8, 9, 10].

**Table 1.** **Classification of cytotoxic drugs and mechanisms of cardiovascular toxicity**.

Drug classification	Representative drugs	Cardiovascular toxicity reactions	Pathological mechanisms
Anthracyclines	Doxorubicin, Epirubicin, Daunorubicin, Idarubicin	Acute Myocardial Injury, Myocarditis, Dilated Cardiomyopathy, Heart Failure, Arrhythmia	ROS Generation, Ca^2+^ Imbalance, Ferroptosis, Mitochondrial Damage, NLRP3 Inflammasome Activation, DNA Topoisomerase IIΒ-Mediated Damage
Alkylating agents	Cyclophosphamide, Cisplatin, Busulfan, Mechlorethamine	Acute Myocardial Necrosis, Pericarditis, Heart Failure, Hypertension, Vascular Injury	Excessive ROS Production, Impaired NO Synthesis, Inflammatory Response, Vascular Endothelial Injury
Antimetabolites	5-fluorouracil, Cytarabine, Methotrexate	Angina Pectoris, Coronary Artery Spasm, Myocardial Ischemia, Arrhythmia	ROS Generation, Mitochondrial Damage, Endothelial Dysfunction
Plant alkaloids	Vincristine, Vinblastine, Paclitaxel, Docetaxel	Arrhythmia, Conduction Block, Hypotension	Microtubule Depolymerization Inhibition, Abnormal Calcium Handling
Others	Bleomycin, Actinomycin D	Pulmonary Hypertension, Right Heart Failure, Arrhythmia	Endothelial Injury, Oxidative Stress, Fibrotic Response

This table summarizes the four major categories of cytotoxic agents commonly used in pediatric hematologic malignancies, their representative drugs, and the predominant mechanisms underlying cardiovascular toxicity. While anthracyclines exert multifactorial cardiotoxic effects involving oxidative stress, Ca^2+^ dysregulation, ferroptosis, DNA damage, and inflammasome activation, alkylating agents primarily cause DNA cross-linking and endothelial injury. Antimetabolites interfere with nucleotide metabolism and mitochondrial function, often leading to coronary vasospasm, whereas plant alkaloids disrupt microtubule dynamics and ion channel function, predisposing to arrhythmias. These pathways converge on cardiomyocyte injury and cardiac dysfunction, but each drug class retains distinct mechanistic signatures. Most available evidence originates from adult cohorts, and pediatric-specific data are notably limited. Mechanistic evidence is predominantly derived from preclinical models; pediatric clinical validation is limited. ROS, reactive oxygen species; DNA, deoxyribonucleic acid; NO, nitric oxide; NLRP3, nod-like receptor family pyrin domain containing 3.

#### 2.1.2 Oxidative Stress and ROS Generation

Cytotoxic agents disrupt the mitochondrial electron transport chain, resulting in excessive generation of reactive oxygen species (ROS)-notably superoxide anion (O_2_•^–^), hydrogen peroxide (H_2_O_2_), and the hydroxyl radical (•OH). The interaction of Surplus ROS with intracellular proteins, lipids, and deoxyribonucleic acid (DNA) has been demonstrated to trigger oxidative stress and subsequent cellular dysfunction [11]. In 2020, Efentakis *et al*. [12] reported that doxorubicin (DOX) can induce alterations in the expression and/or activity of Nicotinamide Adenine Dinucleotide Phosphate (NADPH) oxidases (NOXs). NOX enzymes (NOX1-NOX5) are electrogenic oxidases that drive transmembrane electron transfer-accompanied by compensatory H^+^ currents across the phospholipid bilayer-and catalyze the one-electron reduction of O_2_•^–^. This process promotes the generation and accumulation of ROS in preclinical models, resulting in cardiomyocyte injury; however, pediatric clinical evidence confirming these findings is still lacking. According to Ma *et al*. [13], DOX further activates NOX2 by inducing phosphorylation of p47phox-the cytosolic organizer subunit of the phagocyte NADPH oxidase (NOX2) complex-thereby promoting cardiomyocyte injury. Similar NOX-dependent oxidative signaling has also been documented with alkylating agents-particularly cyclophosphamide via its metabolite acrolein-although the evidence base is smaller than that for anthracyclines [14].

Most evidence supporting NOX-mitochondrial crosstalk arises from murine and *in-vitro* studies, while pediatric clinical data remain scarce. Cytotoxic agents have been demonstrated to induce mitochondrial oxidative stress and ROS generation. ROS-mediated crosstalk between NADPH oxidases (e.g., NOX4) and mitochondria establishes a feed-forward cascade that amplifies oxidative stress, disrupts myocardial energy metabolism, and ultimately leads to cardiac dysfunction [15, 16, 17]. In the study by Aung *et al*. [18], DOX treatment was found to upregulate mitochondrial fission process 1 (Mtfp1) in cardiomyocytes, thereby promoting mitochondrial fission. The suppression of Mtfp1 expression effectively attenuated DOX-induced mitochondrial fission and cardiomyocyte apoptosis, thereby mitigating cardiotoxicity. These preclinical findings indicate that Mtfp1 contributes to DOX-induced mitochondrial fission and subsequent cardiomyocyte apoptosis, highlighting Mtfp1 as a potential therapeutic target to mitigate doxorubicin-associated cardiotoxicity. Recent findings [19] suggest that NOX4-derived ROS not only exacerbate mitochondrial dysfunction but also suppress the activity of antioxidant enzymes (e.g., superoxide dismutase [SOD]), thereby activating the nod-like receptor family pyrin domain containing 3 (NLRP3) inflammasome and establishing a vicious cycle of persistent inflammation and cellular injury that ultimately culminates in cardiomyocyte apoptosis. Antimetabolites, such as cytarabine, have been observed to exacerbate the aforementioned imbalance. This exacerbation is believed to occur as a result of the disruption of nucleotide metabolism and mitochondrial DNA integrity, which in turn leads to secondary oxidative stress [20, 21].

Ferroptosis is an iron-dependent form of regulated cell death characterized by disrupted iron homeostasis and aberrant accumulation of lipid peroxides. This particular form of cell death has attracted growing attention for its pathophysiological involvement in the initiation and progression of DOX (Adriamycin)-induced cardiotoxicity [8, 22]. DOX has been shown to promote the release of mitochondrial ferrous iron (Fe^2+^) and the formation of DOX-Fe^2+^ complexes. This process leads to the accumulation of ROS, which in turn triggers lipid peroxidation-dependent ferroptosis [23]. Concordantly, DOX has been observed to downregulate the cystine/glutamate antiporter system Xc^–^ (Xc^–^) and glutathione peroxidase 4 (GPX4), thereby impairing lipid-peroxide detoxification and further promoting ferroptosis [24]. As demonstrated in studies [25, 26], the ferroptosis inhibitor ferrostatin-1 (Fer-1) has been shown to suppress Fe^2+^-driven lipid peroxidation, thereby substantially mitigating mitochondrial ferroptosis. These observations delineate ferroptosis as a therapeutically actionable driver of injury, highlighting restoration of the Xc^–^-GSH-GPX4 defense, restriction of the labile Fe^2+^ pool, inhibition of lipid-peroxidation mediators, and reinforcement of radical-trapping pathways as potential targets to mitigate cardiotoxicity. It should be noted that ferroptosis-related mechanisms are supported almost exclusively by preclinical evidence, with direct validation in pediatric patients still lacking.

**Fig. 1.**
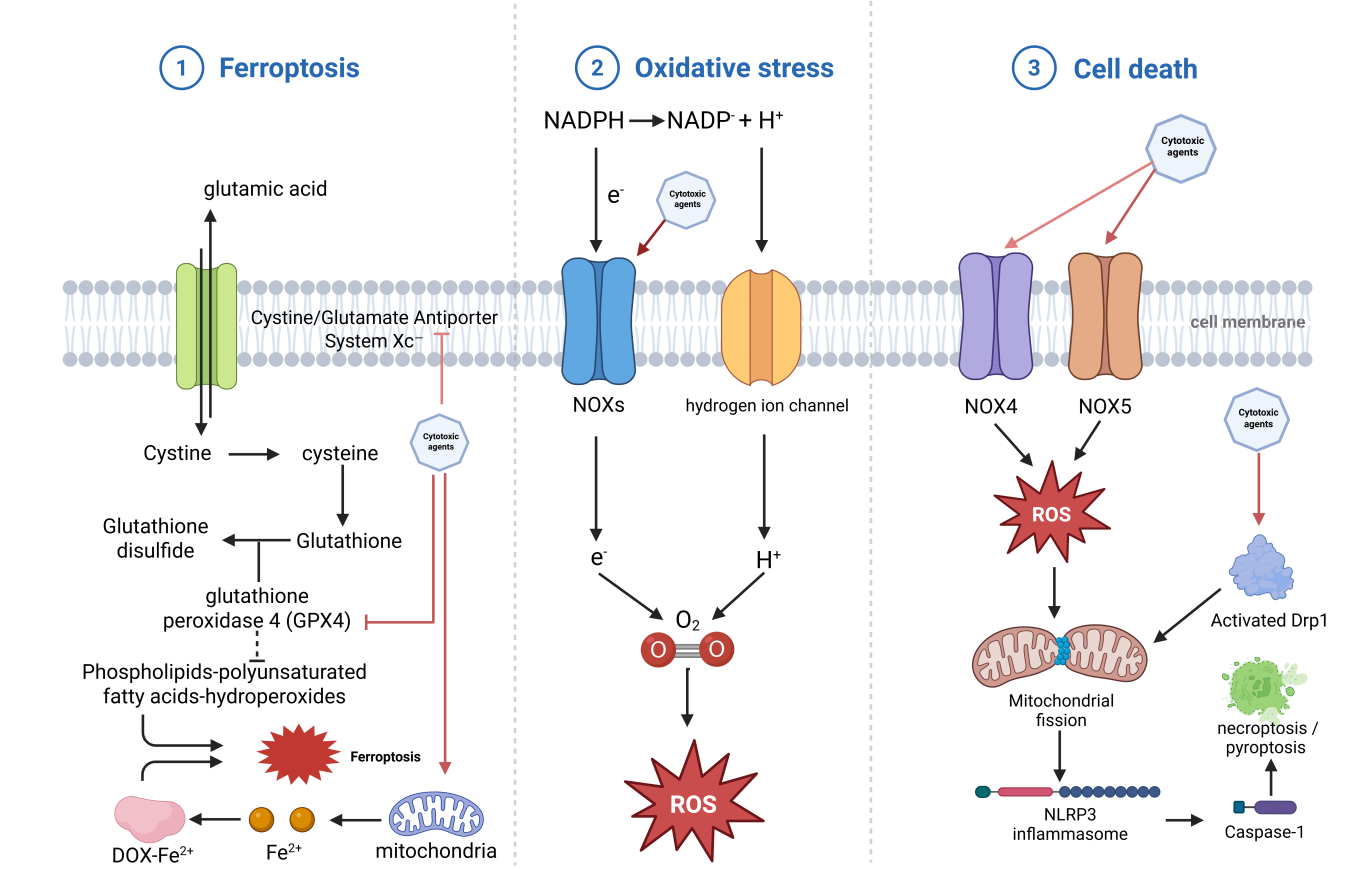
**Oxidative stress-driven ferroptosis and inflammasome activation in cytotoxic therapy-induced cardiotoxicity**. ①Cytotoxic agents suppress the cystine/glutamate antiporter system Xc^–^, impeding cystine uptake and thereby reducing intracellular cysteine availability for glutathione synthesis. Diminished glutathione reserves impair glutathione peroxidase-4 (GPX4) activity, leading to the accumulation of phospholipid polyunsaturated fatty-acid hydroperoxides and iron-dependent lipid peroxidation, ultimately driving ferroptotic cell death; ②Cytotoxic agents enhance the activity of nicotinamide adenine dinucleotide phosphate (NADPH) oxidases (NOXs) and perturb membrane ion flux, promoting electron leakage and hydrogen ion accumulation. These processes augment the production of ROS, which serve as central mediators of treatment-related oxidative injury; ③Upregulation of NOX4 and NOX5 increases ROS generation, while cytotoxic agents concurrently activate dynamin-related protein 1 (Drp1)-dependent mitochondrial fission. Excess ROS and mitochondrial fragmentation converge to activate the NLRP3 inflammasome through caspase-1-dependent signalling, culminating in pyroptosis or necroptosis of cardiomyocytes. Created with BioRender.com (https://www.biorender.com).

Apoptosis is prominently induced in cardiomyocytes and vascular endothelial cells following exposure to cytotoxic agents, largely through ROS-mediated opening of the mitochondrial permeability transition pore, cytochrome c release, and activation of the intrinsic apoptotic pathway [27]. Excess ROS also oxidatively modifies ryanodine receptor 2 (RyR2), leading to Ca^2+^ dysregulation, Ca^2+^/calmodulin-dependent protein kinase II (CaMKII) activation, and subsequent cleavage of procaspase-12, which in turn activates caspase-9 and caspase-3 to drive apoptosis [28]. In parallel, necroptosis and pyroptosis have been implicated as additional regulated cell-death pathways, with DOX-induced upregulation of NOX1/NOX4 and activated dynamin-related protein-1 promoting mitochondrial fission and caspase-1-dependent NLRP3 inflammasome activation, thereby triggering cardiomyocyte pyroptosis [29]. Disruption of antioxidant defences-particularly reductions in GPx, SOD, and catalase-further amplifies ROS-mediated injury [30]. Cytotoxic agents have additionally been shown to increase Keap1 while suppressing nuclear factor erythroid 2-related factor 2 (Nrf2) activity, lowering antioxidant-enzyme expression such as SOD and thereby exacerbating oxidative stress [31]. Plant alkaloids may also weaken antioxidant reserves and enhance oxidative stress, although their relative contribution remains less well defined compared with anthracyclines [9] (Fig. 1).

#### 2.1.3 Calcium Dysregulation

**Fig. 2.**
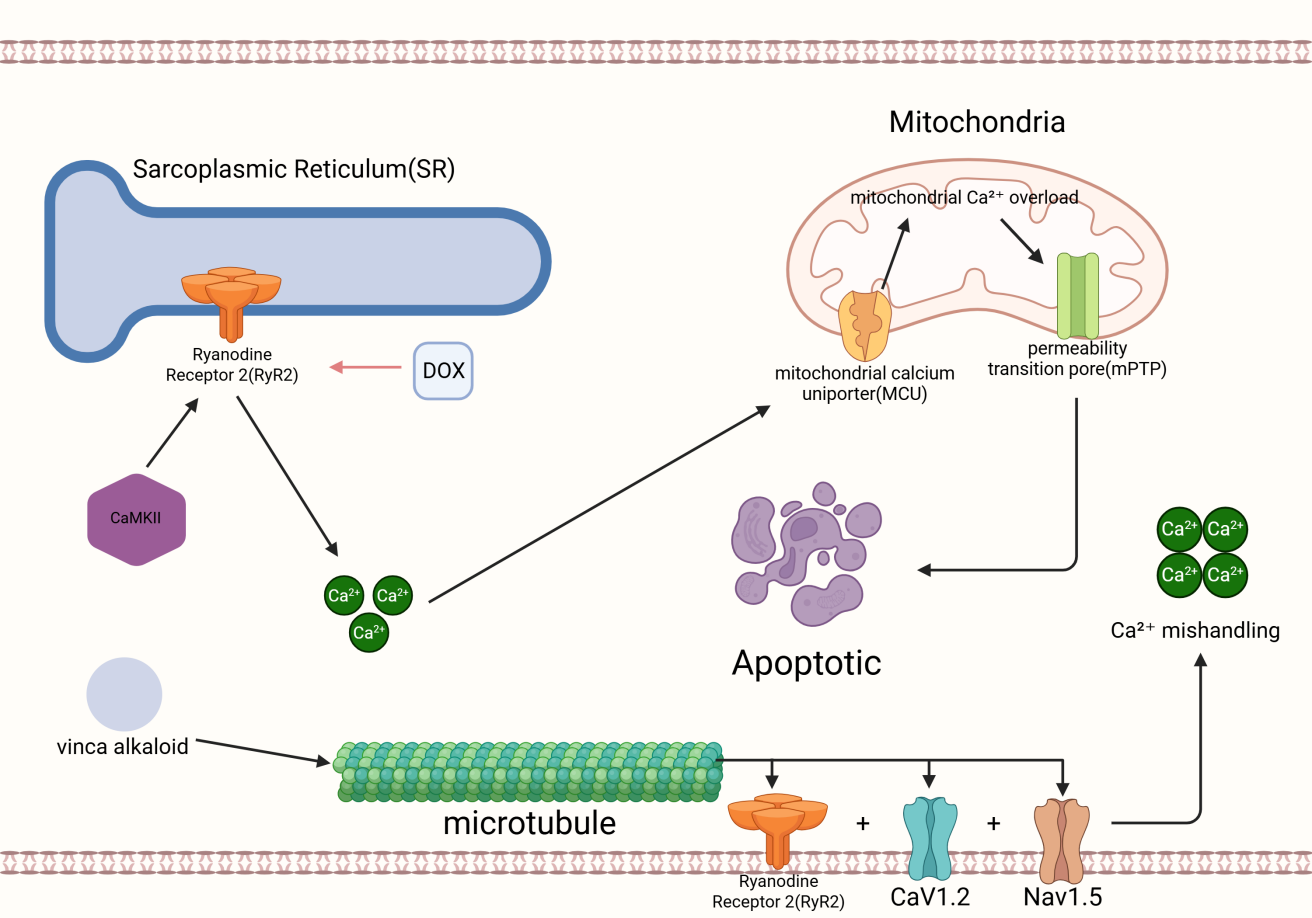
**Calcium dysregulation and mitochondrial injury in doxorubicin-induced cardiotoxicity**. Doxorubicin perturbs sarcoplasmic reticulum (SR) Ca^2+^ handling by promoting CaMKII activation and RyR2 dysfunction, leading to enhanced diastolic SR Ca^2+^ leak and the generation of spontaneous Ca^2+^ waves. Excess cytosolic Ca^2+^ is subsequently taken up by mitochondria through the mitochondrial calcium uniporter (MCU), precipitating mitochondrial Ca^2+^ overload, opening of the mitochondrial permeability transition pore (mPTP), loss of membrane potential, and activation of apoptotic cell-death pathways. In parallel, microtubule-targeting plant alkaloids disrupt the integrity of the microtubule network responsible for trafficking ion-handling proteins-including RyR2, CaV1.2, and Nav1.5-to their correct membrane domains. Impaired microtubule-based transport alters the distribution and surface density of these channels, further exacerbating Ca^2+^ mishandling and contributing to cardiomyocyte injury. Created with BioRender.com (https://www.biorender.com).

In addition to initiating ROS production, dysregulated Ca^2+^ cycling integrates multiple pro-death signals. Pathological CaMKII activation and RyR2 dysfunction, notably CaMKII-dependent phosphorylation at Ser2814 and RyR2 oxidative modifications, enhance diastolic sarcoplasmic reticulum Ca^2+^ leak and spontaneous Ca^2+^ waves. These waves drive mitochondrial Ca^2+^ uptake through the Mitochondrial Calcium Uniporter (MCU) complex, which in turn precipitates permeability transition pore (mPTP) opening, loss of membrane potential, and activation of apoptotic and necrotic programs [32]. In cases of doxorubicin-induced cardiotoxicity, CaMKII signaling worsens injury to cardiomyocytes and interacts with RyR2-mediated Ca^2+^ leakage. This establishes a link between sarcoplasmic reticulum (SR) Ca^2+^ dysregulation and mitochondrial Ca^2+^ overload, which ultimately leads to cell death [33]. Microtubule-targeting plant alkaloids, such as vinca alkaloids and taxanes, may indirectly distort Ca^2+^ homeostasis by disrupting the microtubule network that transports and positions ion-handling proteins, such as Nav1.5, CaV1.2, and RyR2 macromolecular complexes, to specific membrane domains. Microtubule remodeling is increasingly recognized as a factor that determines the delivery, surface density, and excitation-contraction coupling of ion channels in cardiomyocytes [34]. Consistent with this mechanistic model, recent studies have shown that microtubule modifications regulate the distribution and current density of Nav1.5, and that disrupting microtubule-based transport alters the localization of membrane proteins. These findings support the hypothesis that plant alkaloids can indirectly promote Ca^2+^ mishandling by affecting protein trafficking [35]. These observations are based on isolated cardiomyocyte and animal studies, and a direct clinical association with arrhythmia-particularly in pediatric oncology patients-has yet to be demonstrated (Fig. 2).

#### 2.1.4 Endothelial Cell Injury

In addition to directly damaging cardiomyocytes, cytotoxic drugs often impair vascular endothelial function. This contributes to acute and chronic cardiovascular complications. High-dose alkylating agents, notably cyclophosphamide and cisplatin, form DNA inter- and intrastrand crosslinks, overwhelming endothelial DNA repair capacity and activating p53-dependent checkpoints and apoptosis in endothelial cells (ECs) [36]. *In vitro* and *in vivo* data demonstrate that cisplatin directly triggers EC apoptosis and anti-angiogenic injury. In contrast, cyclophosphamide and its metabolite acrolein injure ECs by promoting detachment, death, and thrombogenicity [37]. This endothelial cell loss leads to capillary rarefaction and microvascular dysfunction, resulting in reduced NO bioavailability, impaired vasoreactivity, and diminished tissue perfusion. This provides a mechanistic bridge from alkylator exposure to ischemia and subsequent cardiac dysfunction [38].

Endothelial activation is a critical factor that leads to a pro-inflammatory and pro-thrombotic vascular state. It has been demonstrated that endothelial cells exhibit an increase in adhesion molecules (vascular cell adhesion molecule-1 [VCAM-1] and intercellular adhesion molecule-1 [ICAM-1]) in response to redox/inflammatory stress induced by cytotoxic therapy. These adhesion molecules play a crucial role in mediating leukocyte tethering, firm adhesion, and transendothelial migration, which can lead to the seeding of vascular inflammation and microvascular injury. Concurrent release of Weibel-Palade–derived P-selectin/vWF, reduced NO bioavailability, and tissue-factor/plasminogen activator inhibitor-1 (PAI-1) shifts further propagate platelet activation and coagulation, linking endothelial activation to thrombosis [39]. Clinically, this endothelial program manifests as impaired endothelium-dependent vasodilation and a pro-thrombotic phenotype after anthracyclines and alkylating agents. Human studies and guidelines in cardio-oncology recognize endothelial dysfunction as a key intermediate of cancer-therapy vascular toxicity [40]. Fluoropyrimidines have been demonstrated to induce a vasospastic response. 5-fluorouracil (5-FU) has been extensively documented to induce coronary vasospasm, frequently manifesting as angina or ischemia despite the absence of angiographic abnormalities in the epicardial arteries. Expert analyses and guidelines underscore vasospasm and endothelial injury as the predominant mechanisms [41]. It is important to emphasize that these data are derived predominantly from adult oncology cohorts, as pediatric-specific evidence remains exceedingly limited.

**Fig. 3.**
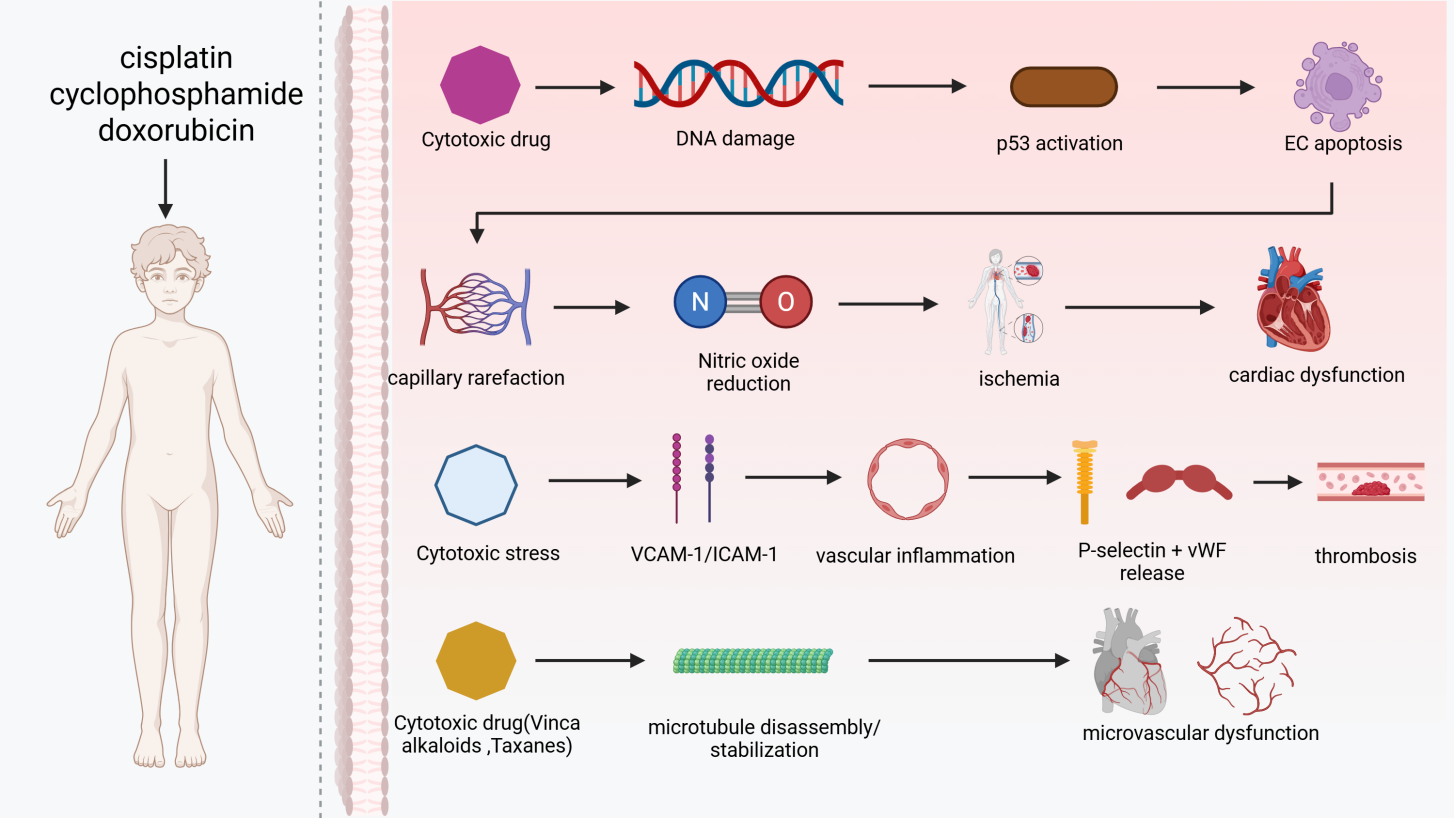
**Endothelial injury and microvascular dysfunction induced by cytotoxic therapies**. Cisplatin, cyclophosphamide and doxorubicin initiate endothelial toxicity by inducing DNA inter- and intrastrand crosslinks, activating p53 signalling, and triggering apoptosis of endothelial cells (ECs). Loss of endothelial integrity leads to capillary rarefaction, reduced NO bioavailability, impaired vasoreactivity and downstream myocardial ischaemia, ultimately contributing to cardiac dysfunction. In parallel, cytotoxic therapy promotes endothelial activation characterised by increased vascular cell adhesion molecule-1 (VCAM-1) and intercellular adhesion molecule-1 (ICAM-1) expression, facilitating leukocyte adhesion and vascular inflammation. Concomitant release of Weibel-Palade-derived P-selectin and von Willebrand factor (vWF) augments platelet activation and thrombosis formation. Microtubule-targeting agents such as vinca alkaloids and taxanes disrupt endothelial cytoskeletal structure through microtubule disassembly or hyperstabilisation, impairing endothelial migration and repair, and thereby promoting microvascular dysfunction. Created with BioRender.com (https://www.biorender.com).

Chronic endothelial dysfunction after cytotoxic therapy has been shown to accelerate atherogenesis and adverse vascular remodeling. This process occurs via sustained nitric oxide (NO) depletion, oxidative stress, and pro-inflammatory/pro-thrombotic signaling. Over time, this predisposes survivors to premature coronary artery disease (CAD). The effect is amplified in those who also received chest radiotherapy [39]. Platinum and alkylating regimens (e.g., cisplatin, cyclophosphamide) contribute to this phenotype by causing persistent endothelial injury and dysfunction. Epidemiologic and mechanistic data in testicular cancer cohorts link platinum exposure to early atherosclerosis and ischemic events [42]. Plant alkaloids that target microtubules have been shown to disrupt endothelial cytoskeletal integrity and barrier/migratory function. Specifically, vinca alkaloids have been observed to disrupt microtubule assembly and exert vascular-disrupting effects, while taxanes have been demonstrated to stabilize microtubules, impeding endothelial migration and repair. This collaborative effect of taxanes and vinca alkaloids has been identified as a contributing factor to vascular fragility and microvascular dysfunction [43, 44, 45] (Fig. 3).

### 2.2 RT

RT remains an essential component in the treatment of pediatric hematologic malignancies [46]. However, even with meticulous planning, the procedure can result in exposure of the heart and great vessels to ionizing radiation. Preliminary clinical observations have indicated that in adult cancer cohorts, particularly survivors of breast cancer and Hodgkin lymphoma, chest radiotherapy has been associated with an approximately 4–6-fold increase in the risk of coronary artery disease, with risk rising in an almost linear fashion with increasing cardiac radiation dose [47]. Pediatric dose-response data for radiation-associated coronary disease remain far more limited, and current assumptions about susceptibility are largely extrapolated from these adult observations. This exposure can trigger a cascade of events, beginning with DNA injury and oxidative stress and culminating in inflammation, fibrosis, and clinically evident cardiovascular disease (CVD) [48]. Acute toxicities (e.g., pericarditis, arrhythmias) may manifest during or shortly after RT, whereas chronic complications (e.g., coronary artery disease, valvular disease, heart failure) generally emerge years to decades later, with an increased risk associated with cardiac dose and younger age at exposure [48, 49, 50, 51, 52, 53].

**Fig. 4.**
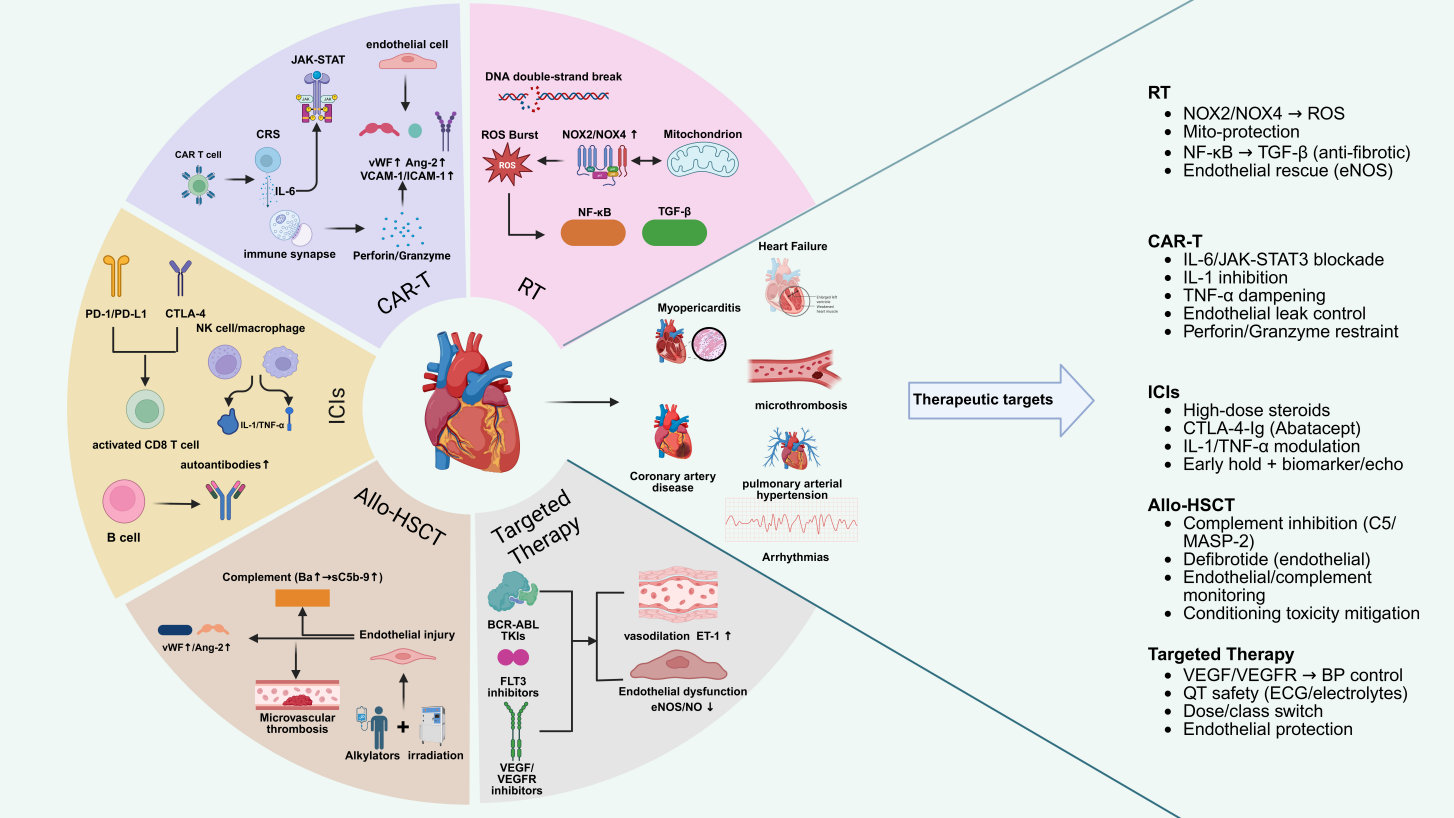
**Mechanistic landscape of cancer therapy–related cardiovascular toxicity in pediatric hematologic malignancies**. RT (DNA double-strand breaks → persistent ROS with NOX2/NOX4-mitochondria crosstalk → nuclear factor kappa-light-chain-enhancer of activated b cells (NF-κB)/transforming growth factor-beta (TGF-β) -driven fibrosis → pericarditis/arrhythmias [acute] and coronary artery disease (CAD)/valvulopathy/heart failure [late]); chimeric antigen receptor T-cell (CAR-T) (interleukin-6 [IL-6]/janus kinase [JAK]-signal transducer and activator of transcription [STAT] -dominated cytokine-release syndrome [CRS] with endothelial activation, capillary leak and hypotension; parallel perforin/granzyme immune-synapse injury leading to myocarditis/arrhythmias); immune checkpoint inhibitors (ICIs) (programmed cell death protein 1 [PD-1]/programmed death-ligand 1 [PD-L1] & cytotoxic t-lymphocyte associated protein 4 [CTLA-4] blockade unleashing autoreactive T cells, aided by B/natural killer (NK)/macrophage effectors, causing lymphocytic myocarditis and conduction disease); allogeneic hematopoietic stem-cell transplantation (Allo-HSCT) (myeloablative conditioning and immune incompatibility provoking endothelial syndromes via complement activation [Ba, sC5b-9], culminating in hypertension, thrombosis, and cardiac dysfunction); and Targeted agents (class-patterned endothelial dysfunction with endothelial nitric oxide synthase (eNOS)/NO↓ and endothelin-1 (ET-1)↑, ion-channel and mitochondrial stress, yielding hypertension/pulmonary arterial hypertension (PAH), arterial events, corrected QT interval (QTc) prolongation, and heart failure). Created with BioRender.com (https://www.biorender.com).

Ionizing radiation has been demonstrated to induce direct and indirect DNA damage in cardiomyocytes, endothelial cells, and fibroblasts [54, 55]. The latter effect is primarily mediated by water radiolysis and ROS [54]. Sustained oxidative stress is amplified by mitochondrial dysfunction and NADPH oxidase (NOX2/NOX4) activation within cardiac and vascular tissues, lowering nitric-oxide bioavailability, promoting endothelial activation, and driving lipid, protein, and DNA oxidation [5, 56]. Downstream, pro-inflammatory signaling (e.g., nuclear factor kappa-light-chain-enhancer of activated b cells [NF-κB]) and cytokine release recruit immune cells and stimulate myofibroblast transdifferentiation with excess extracellular-matrix deposition-hallmarks of radiation-induced myocardial and perivascular fibrosis [57]. These biologic programs establish a correlation between acute injury and late structural remodeling, as well as diastolic and systolic dysfunction [58, 59]. In children, smaller cardiac volumes and ongoing myocardial development heighten susceptibility to dose-volume effects, particularly in the coronary ostia, cardiac valves, and right-sided chambers. Furthermore, there is evidence that RT-induced cell damage can trigger the body’s inflammatory response, resulting in the release of a multitude of inflammatory factors [60]. These inflammatory factors may play a pivotal role in the development and progression of atherosclerosis and coronary atherosclerotic heart disease. Long-term pediatric survivor studies from the CCSS and PENTEC initiatives demonstrate that childhood cardiac radiation exposure with mean heart doses ≥10 Gy is associated with a dose-dependent increase in late cardiac events, including coronary artery disease and valvular dysfunction, even at what would traditionally be considered modest dose levels [51, 52, 61] (Fig. 4).

### 2.3 CAR-T

CAR-T therapy has emerged as a notable treatment modality for Pediatric hematological malignancies. In pivotal studies of CD19-directed CAR-T therapy (tisagenlecleucel), overall remission rates of almost 90% have been reported in children and young adults with relapsed or refractory B-cell precursor acute lymphoblastic leukaemia [62]. However, its robust immune activation can result in cardiovascular injury. From a mechanistic perspective, in both adult and pediatric reports, cardiotoxicity is primarily attributable to cytokine-release syndrome (CRS), which is characterized by interleukin-6 (IL-6)/janus kinase (JAK)-signal transducer and activator of transcription (STAT) signaling and subsequent endothelial activation [63, 64, 65]. Although mechanistic detail specific to pediatric populations remains limited, CRS-driven IL-6/janus kinase (JAK)-signal transducer and activator of transcription (STAT) signalling and endothelial activation appear to underpin most cases. Cell-mediated cytotoxicity (perforin/granzyme) and immune-cell infiltration can drive myocarditis in a subset [66]. Furthermore, a study published by Barachini *et al*. in 2024 [9] highlighted that the interaction between CAR-T cells and tumour cells results in the release of perforin and granzymes, which in turn induce tumour cell apoptosis. Preclinical and adult case-based evidence suggests that perforin and granzymes may contribute to bystander myocardial injury, although pediatric-specific data on CAR-T-associated myocarditis remain scarce, thereby potentially precipitating myocarditis and heart failure.

Antigen engagement has been demonstrated to trigger rapid T-cell expansion and macrophage/monocyte activation, accompanied by surges of IL-6, interleukin-6 (IL-1), interferon-gamma (IFN-γ), and tumor necrosis factor-alpha (TNF-α) [67]. IL-6 signals through the gp130/JAK–STAT3 pathway, inducing nitric-oxide synthase and mitochondrial stress in cardiomyocytes, which in turn depresses contractility (“myocardial stunning”) [68]. Concurrently, the endothelium transitions to a pro-inflammatory, pro-thrombotic state (elevated von Willebrand factor, increased angiopoietin-2, adhesion molecule up-regulation), resulting in capillary leak, interstitial oedema, microvascular thrombosis, and impaired coronary microcirculation [69]. The aforementioned changes have been shown to be associated with systemic hypotension (driven by vasoplegia and capillary leak), supply-demand mismatch, and troponin release in the setting of CRS during CAR-T therapy [70]. Clinically relevant myocarditis following CAR-T therapy remains uncommon in children, and most cardiovascular events arise in the context of high-grade cytokine-release syndrome rather than isolated immune-mediated myocarditis. Furthermore, severity of myocardial injury has been demonstrated to closely track CRS grade and cytokine burden [71]. Cytokine-induced electrical instability (for example, via STAT-dependent channel modulation and sympathetic surge) provides an arrhythmogenic substrate [72]. These mechanistic insights are derived predominantly from adult or mixed-age cohorts, with pediatric cardiac validation remaining limited.

At the immune synapse, activated CAR-T cells discharge perforin and granzyme B to lyse tumour targets [73]. In a highly inflamed milieu, bystander injury may occur via spillover cytotoxic granules, collateral killing of stressed cardiomyocytes, and myocardial infiltration by activated T cells [74]. Histopathological analysis of reported cases reveals lymphocytic myocarditis, accompanied by myocyte necrosis, indicative of cell-mediated cytotoxic activity [75]. The potential contribution of rare off-target or cross-reactive recognition mechanisms should be considered [76]. In the downstream process, the activation of the inflammasome and amplification of myeloid cells (IL-1/TNF-α) can lead to escalation of necro-inflammatory damage [77] (Fig. 4).

### 2.4 Immune Checkpoint Inhibitors (ICIs) 

At present, a considerable number of types of ICIs have been approved by the U.S. Food and Drug Administration (FDA) for clinical treatment [78]. In adult cohorts, the incidence of ICI-related myocarditis is approximately 0.06% with programmed cell death protein 1 (PD-1) monotherapy, rising to around 0.27% with combined nivolumab-ipilimumab therapy (Johnson *et al*., NEJM 2016 [79]; *p* < 0.001). The incidence in pediatric populations is unknown but is presumed to be lower [79, 80]. However, in recent years, several case reports have indicated that some patients have experienced serious adverse cardiac events, such as arrhythmias and myocarditis, during the use of such drugs [79, 81, 82, 83, 84, 85]. Consequently, as the application of ICIs therapy in the pediatric population continues to expand, its potential risk of causing serious cardiovascular damage urgently needs to be paid close attention. According to the latest literature reports [86], ICIs act by blocking the PD-1/programmed death-ligand 1 (PD-L1) and cytotoxic t-lymphocyte associated protein 4 (CTLA-4) signaling pathways, thus relieving the immune suppression of T cells and enhancing the immune response. However, excessive activation of T cells may cause them to misidentify and attack normal myocardial cells, thereby inducing myocarditis.

Physiological PD-1/PD-L1 and CTLA-4 signalling has been demonstrated to restrict the proliferation of autoreactive T cells within peripheral tissues [87]. Therapeutic checkpoint blockade has been shown to remove these brakes, thereby enabling clonal expansion and effector differentiation of both CD8^+^ (and CD4^+^) T cells, which exhibit cross-reactivity to cardiac antigens (e.g., cardiac myosin) and dense myocardial infiltration [88]. Murine and translational studies demonstrate that anti-PD-1/PD-L1 or dual PD-1/CTLA-4 inhibition precipitates lymphocytic myocarditis with myocyte necrosis; tissue transcriptomics show T-cell activation programs and macrophage amplification [89]. From a clinical perspective, this mechanism elucidates the early timing of symptoms, the elevated troponin levels, the presence of conduction disease, and the frequent response to high-dose corticosteroids [90]. These mechanisms are derived largely from adult myocarditis models, with pediatric mechanistic data remaining extremely limited.

Activated cytotoxic lymphocytes at the cardiomyocyte “immune synapse” deploy perforin and granzyme B, inducing pore-formation and apoptotic/necrotic death of stressed myocytes [91]. Bystander injury can occur when systemic inflammation is high (e.g., concurrent cytokine surges) [92]. The pathology of the reported cases has been shown to be consistent with lymphocytic myocarditis, with myocyte dropout, which is consistent with cell-mediated cytotoxic activity [93]. These processes are potentiated by macrophage-derived cytokines (IL-1, TNF-α) and may be more frequent with combined checkpoint blockade [93].

Furthermore, other studies [94, 95] have indicated that Preclinical and adult translational studies suggest that ICIs modulate the activity of B cells, natural killer (NK) cells, and macrophages; however, whether these pathways contribute to cardiovascular injury in pediatric patients remains unknown. Consequently, the early recognition and definitive diagnosis of heart failure should be based on an integrated assessment comprising clinical evaluation, electrocardiography, cardiac biomarkers, and cardiac imaging. Prompt discontinuation of ICIs and early initiation of immunosuppression are recommended based on experience in adult myocarditis [96, 97], although pediatric evidence remains confined to case-based reports (Fig. 4).

### 2.5 Allo-HSCT

Allo-HSCT is a potentially curative therapy for pediatric hematologic malignancies, but it confers both early and late cardiovascular (CV) risks that arise from conditioning toxicity, endothelial injury syndromes, immune dysregulation, and prolonged immunosuppression; recent American Heart Association (AHA) scientific statements and Chinese national consensus documents alike emphasize life-long, risk-adapted CV surveillance in survivors [98].

Myeloablative or intensified conditioning regimens can injure cardiomyocytes and vascular endothelium through DNA damage, oxidative stress, and mitochondrial dysfunction, which can result in arrhythmias, heart failure, thrombotic microangiopathy (TA-TMA), and sinusoidal obstruction syndrome (SOS) [99]. These mechanisms are derived largely from adult HSCT cohorts and preclinical studies, with pediatric-specific mechanistic data remaining limited, although similar biological susceptibilities are generally presumed. Pediatric and adult reviews concur that conditioning intensity and chest/total-body RT dose scale CV risk, and these exposures should be incorporated into pre-HSCT risk stratification and post-HSCT follow-up plans [99].

The period immediately preceding and subsequent to transplantation is characterised by a heightened inflammatory response, characterised by elevated levels of cytokines. This heightened response is accompanied by activation of the endothelial cells, as evidenced by an increase in von Willebrand factor (vWF) and complement activation [88]. These changes can precipitate a series of adverse outcomes, including capillary leak, microthrombosis, and myocardial stress. These events contribute to the occurrence of early cardiovascular events and subsequent atherosclerotic remodelling. The activation of the complement pathway is increasingly recognised in pediatric endothelial complications; elevated complement Ba and other markers predict the risk of thrombotic acute graft-versus-host disease (TA-TMA/SOS) following haematopoietic stem cell transplantation (HSCT) [100].

Immune incompatibility (e.g., HLA mismatch or unrelated donors) and acute Graft-Versus-Host Disease (GVHD) have been demonstrated to amplify endothelial-injury syndromes (TA-TMA, SOS) and increase non-relapse morbidity, partly through intensified inflammation and calcineurin-inhibitor exposure [101]. In the field of pediatric research, prominent and reliable sources have identified unrelated or Human Leukocyte Antigen (HLA)-mismatched donors and myeloablative conditioning as factors that increase the likelihood of hepatic sinusoidal and vascular complications following HSCT [100]. Genetic susceptibility in the complement/endothelial pathways (in both patients and donors) further modifies risk, thus supporting the implementation of precision monitoring in children [102] (Fig. 4).

### 2.6 Small-Molecule Targeted Therapy

The field of tumor genomics and molecular pathology has undergone rapid development in recent decades, with substantial advances being made in the area of small-molecule targeted therapeutics. The utilisation of small-molecule targeted agents in the treatment of pediatric hematological malignancies, with a particular focus on BCR-ABL tyrosine kinase inhibitors (TKIs), Fms-like tyrosine kinase 3 (FLT3) inhibitors, and vascular endothelial growth factor (VEGF)/vascular endothelial growth factor receptor (VEGFR) inhibitors, gives rise to a range of cardiovascular toxicities. These include endothelial dysfunction, hypertension, corrected QT interval (QTc)-interval prolongation/arrhythmia, heart failure, and arterial/venous vascular events [103].

In 2001, the U.S. Food and Drug Administration (FDA) approved the TKI imatinib for the treatment of adult chronic myeloid leukemia (CML), with the indication subsequently extended to pediatric CML in 2003 [104]. In adults, TKI therapy carries a well-characterised risk of cardiovascular adverse events. Pediatric data are far more limited, although case-based evidence suggests that similar pathways-endoplasmic reticulum (ER) stress, mitochondrial dysfunction, and metabolic reprogramming-may contribute to toxicity in younger patients. For instance, the first-generation TKI imatinib has been documented to induce endoplasmic reticulum stress, resulting in mitochondrial dysfunction and cardiomyocyte apoptosis; conversely, the third-generation TKI ponatinib has the capacity to inhibit protein kinase B (AKT) and extracellular signal-regulated kinase (ERK) signalling, perturb cardiomyocyte metabolism, and has been associated with hypertension, heart failure, arrhythmias, atherosclerosis, and other cardiometabolic abnormalities [104].

Inhibition of VEGF signalling has been demonstrated to reduce endothelial nitric oxide bioavailability, increase endothelin-1, and promote microvascular rarefaction, thereby producing a dose-dependent rise in systemic blood pressure and afterload [105]. Pharmacovigilance, clinical, and preclinical syntheses provide further support for an immune-mediated and endothelial-injury signature as a potential underlying mechanism of anti-angiogenic-induced hypertension and vascular events [106]. Children may exhibit heightened blood-pressure sensitivity to VEGF/VEGFR inhibition owing to smaller arterial compliance and ongoing vascular development. In the context of adult AML, sorafenib has been demonstrated in studies to induce endothelial dysfunction through the inhibition of the VEGF signalling pathway and the platelet-derived growth factor receptor (PDGFR) signalling cascade [107]. At present, the question of whether small-molecule targeted therapies exhibit differential risk profiles between adult and pediatric patients remains unanswered.

Compared with adults, children receiving TKIs or anti-angiogenic agents differ in pharmacokinetics, baseline cardiovascular physiology, and long-term exposure horizons. Developmental differences in vascular tone, endothelial function, and ion-channel maturation may shape toxicity profiles; however, robust pediatric cardiotoxicity datasets for small-molecule agents remain sparse. There is an urgent need for systematic pediatric registries and exposure-response studies to define risk more precisely (Fig. 4).

### 2.7 Cardiac Monitoring During Therapy: A Risk-Adapted Workflow for Pediatric Patients

**Fig. 5.**
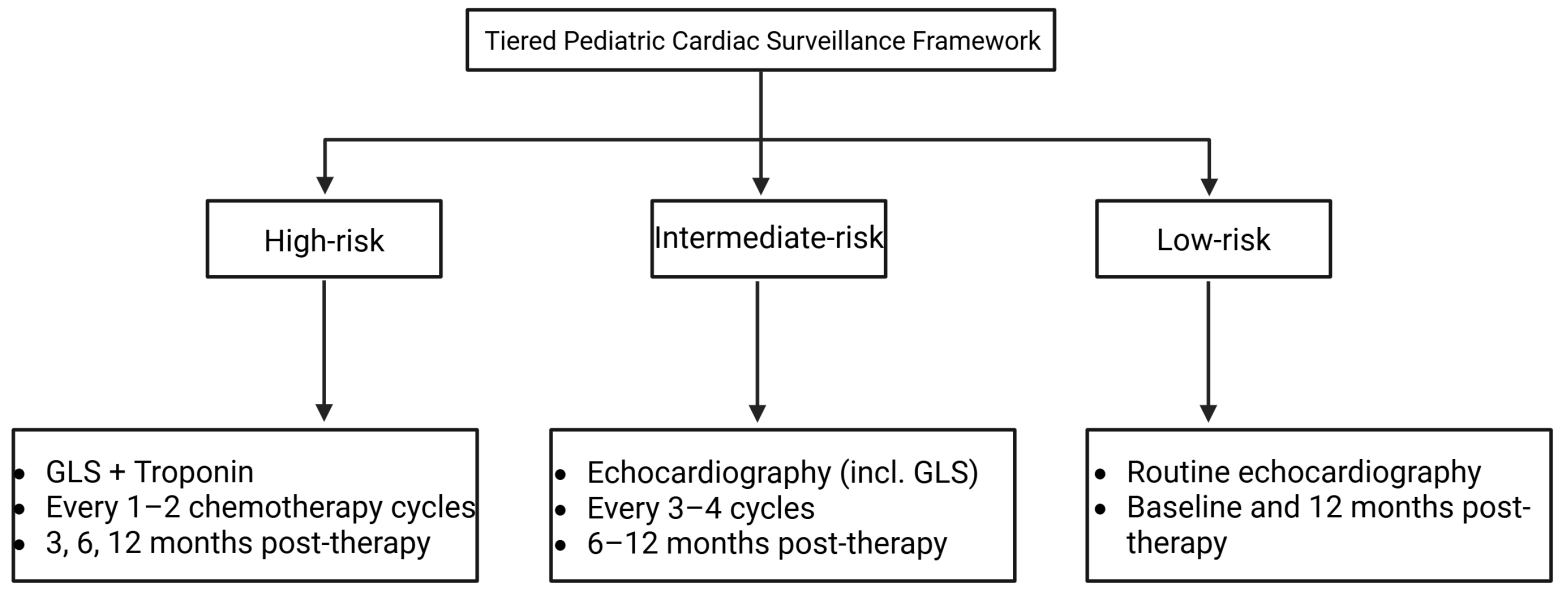
**Risk-adapted cardiac monitoring framework for pediatric cardiotoxic cancer therapies**. This framework integrates treatment- and patient-specific risk stratification with multimodal monitoring-Global longitudinal strain (GLS), cardiac biomarkers, Electrocardiogram (ECG), and Cardiovascular magnetic resonance (CMR) -to guide surveillance during cardiotoxic therapy. High-, intermediate-, and low-risk tiers define monitoring intensity, while actionable thresholds (≥15% GLS reduction, biomarker elevation, left-ventricular ejection fraction (LVEF) decline, or arrhythmia) prompt intensified follow-up and early cardioprotective intervention. This ≥15% reduction in GLS is based on adult consensus recommendations and has not yet been prospectively validated in pediatric populations; accordingly, its application in children should be interpreted with caution. Created with BioRender.com (https://www.biorender.com).

Building on the multimodal mechanisms outlined above, the surveillance targets map to quantifiable domains. These include: Global longitudinal strain (GLS) to detect early systolic impairment; Biomarkers of myocardial injury and haemodynamic stress (troponin and natriuretic peptides); Electrocardiogram (ECG) to detect conduction disease and arrhythmias; Cardiovascular magnetic resonance (CMR) for tissue characterisation (oedema/fibrosis) integrating. These tools into pediatric oncology care through a structured ‘risk stratification→trigger thresholds→intervention’ pathway, tailored to exposure profiles and individual risk, is now widely advocated across multidisciplinary guidance [5] (Fig. 5).

Baseline risk stratification should document regimen-level exposures-including planned cumulative anthracycline dose, thoracic or mediastinal radiotherapy (field, dose, fractionation), Allo-HSCT conditioning, and high-risk combinations such as anthracycline + RT or ICIs + other immune/targeted agents-while integrating patient-level variables (congenital/prior heart disease, blood-pressure percentiles, body-mass index, family history, and modifiable risks) to define a monitoring tier and cadence [5]. At baseline, a standardized evaluation should include history and physical examination with blood pressure, 12-lead ECG (rhythm, intervals, QTc), transthoracic echocardiography with left-ventricular ejection fraction (LVEF) and GLS using laboratory-specific reference values, serum troponin and natriuretic peptides, and CMR when echocardiographic quality or tissue characterization (edema/fibrosis) may alter management [108]. Embedding a checklist of exposures, patient factors, and planned assessments within the oncology protocol facilitates coordination with cardio-oncology teams [5].

On-treatment surveillance is stratified by exposure and risk. High-risk regimens (anthracycline-intensive, overlapping mediastinal RT, Allo-HSCT, or high-risk combinations) require GLS-based echocardiography at cumulative-dose milestones, peri-cycle biomarkers, and early ECG/CMR when discordant or when myocarditis, edema, or fibrosis is suspected [5]. Standard-risk regimens undergo interval imaging at protocol landmarks, blood-pressure monitoring each visit, and prompt evaluation for new symptoms or biomarker elevation [109]. Action thresholds-clinically meaningful GLS decline, LVEF reduction beyond test-retest variability, dynamic troponin/natriuretic peptides rise, or notable arrhythmia-should prompt joint cardio-oncology review, oncologic dose adjustment, and cardioprotective therapy (angiotensin-converting enzyme inhibitor or β-blocker when indicated) [110].

## 3. Limitations

This narrative review is potentially susceptible to publication bias, in which studies with positive or mechanistically compelling results are more frequently reported, as well as to selection bias intrinsic to non-systematic evidence synthesis. For several emerging treatment modalities-particularly CAR-T therapy, immune checkpoint inhibitors, and small-molecule targeted agents-pediatric-specific evidence remains sparse, necessitating cautious extrapolation from adult studies. Substantial heterogeneity across published reports in both incidence and effect size further limits direct comparability, and quantitative estimates should be interpreted in the methodological context of their respective source cohorts. Finally, the proposed surveillance strategies and mechanistic therapeutic targets will require prospective validation in pediatric oncology populations before clinical implementation.

## 4. Conclusions

Cardiovascular toxicity remains a major determinant of long-term outcomes in children with hematological malignancies. Unlike adults, pediatric patients face unique developmental vulnerabilities, longer exposure horizons, and distinct cardiometabolic trajectories. By integrating mechanistic pathways across chemotherapy, radiotherapy, CAR-T therapy, ICIs, Allo-HSCT, and Small-molecule targeted therapy, this review highlights convergent biological nodes that may serve as actionable entry points for cardioprotection in pediatric practice.

Building on mechanistic insights, several candidate cardioprotective targets emerge across treatment modalities. For cytotoxic chemotherapy, promising strategies include inhibition of the NOX-dynamin-related protein 1 (Drp1)-NLRP3 axis and NOX4, modulation of Mtfp1 to limit mitochondrial fission, suppression of transcription factor EB (TFEB) and poly(ADP-ribose) polymerase (PARP) signalling, and restoration of the Xc^–^-GSH-GPX4 antioxidant axis to prevent ferroptosis. Several preclinical agents have shown promise in mitigating CTR-CVT. NOX inhibitors (e.g., GKT137831) have been shown to attenuate ROS generation and suppress NLRP3 activation in DOX-exposed murine cardiomyocytes. Ferroptosis inhibitors (ferrostatin-1 and liproxstatin-1) prevent lipid-peroxide accumulation and enhance mitochondrial membrane stability in pediatric-relevant models of DOX exposure. GPX4 stabilisers represent emerging candidates for clinical translation. Although none of these compounds has yet advanced to pediatric cardio-oncology trials, their mechanistic convergence highlights a set of feasible translational directions. These mechanism-guided strategies provide a rational framework to prioritize cardioprotective trials that preserve anticancer efficacy while improving long-term cardiovascular outcomes in children.

Future research and clinical management should pursue three complementary priorities. First, deepen mechanistic investigation across modalities (cytotoxic agents, RT, CAR-T, ICIs, Allo-HSCT, and targeted agents) to map how oxidative stress, calcium dysregulation, regulated cell-death programs (apoptosis, necroptosis, pyroptosis), immune/inflammatory signalling, and endothelial dysfunction produce shared and treatment-specific cardiac and vascular injury. Such mechanistic clarity will underpin rational cardioprotective design. Second, prioritise translational development of targeted interventions-NOX-Drp1-NLRP3 axis inhibitors, NOX4 inhibitors, Mtfp1 modulators, TFEB/PARP inhibitors, ferroptosis blockers, CaMKII modulators, and endothelial-protective agents-and test their ability to mitigate cardiotoxicity without impairing antitumour efficacy. Third, A tiered pediatric surveillance framework may be considered. High-risk patients (e.g., cumulative DOX ≥250 mg/m^2^, radiotherapy mean heart dose ≥15 Gy, prior HSCT): GLS and troponin every 1–2 chemotherapy cycles, and at 3, 6, and 12 months following completion of therapy. Intermediate-risk patients: Echocardiography (including GLS) every 3–4 chemotherapy cycles, with follow-up imaging at 6–12 months post-therapy. Low-risk patients: Routine echocardiography at baseline and at 12 months post-therapy. Actionable threshold: A relative GLS reduction of ≥15% or a troponin increase above assay-specific upper normal limits should prompt intensified surveillance or consideration of cardioprotective therapy.

A next-generation risk model for CTR-CVT in children could integrate. Clinical variables: age, sex, baseline GLS, cumulative anthracycline dose, radiotherapy mean heart dose (MHD), and HSCT conditioning intensity. Biomarkers: high-sensitivity troponin and NT-proBNP. Genetics: RARG, SLC28A3, and TOP2B polymorphisms associated with anthracycline-induced injury. Therapy-specific exposures: CAR-T CRS grade, ICI regimen, and TKI class. Machine-learning-based models (e.g., random forests and gradient boosting) may enable dynamic, individualised risk estimation, with validation achievable through existing pediatric survivorship cohorts such as CCSS and St. Jude LIFE.

In conclusion, it is imperative to address cardiotoxicity in the context of pediatric hematological malignancies through an integrated approach that encompasses mechanistic discovery, preventive pharmacology, precision risk stratification, and long-term survivorship care. The integration of advancements in molecular cardiology with those in oncology facilitates the development of strategies that ensure the preservation of the cardiovascular system without compromising antitumor efficacy. Ultimately, integrating mechanistic discovery with pediatric-tailored surveillance, early cardioprotection, and data-driven risk prediction will be essential to ensure that children cured of hematological malignancies can also achieve long-term cardiovascular health.

## References

[1] Steliarova-Foucher E, Colombet M, Ries LAG, Moreno F, Dolya A, Bray F (2017). International incidence of childhood cancer, 2001-10: a population-based registry study. *The Lancet. Oncology*.

[2] Hu Y, Liu Y, Fu J, Liu Y, Wang H, Song Y (2024). Global, regional, and national burden of acute lymphoblastic leukemia in children: Epidemiological trends analysis from 1990 to 2021. *iScience*.

[3] Ward E, DeSantis C, Robbins A, Kohler B, Jemal A (2014). Childhood and adolescent cancer statistics, 2014. *CA: A Cancer Journal for Clinicians*.

[4] Gibson TM, Mostoufi-Moab S, Stratton KL, Leisenring WM, Barnea D, Chow EJ (2018). Temporal patterns in the risk of chronic health conditions in survivors of childhood cancer diagnosed 1970-99: a report from the Childhood Cancer Survivor Study cohort. *The Lancet. Oncology*.

[5] Lyon AR, López-Fernández T, Couch LS, Asteggiano R, Aznar MC, Bergler-Klein J (2022). 2022 ESC Guidelines on cardio-oncology developed in collaboration with the European Hematology Association (EHA), the European Society for Therapeutic Radiology and Oncology (ESTRO) and the International Cardio-Oncology Society (IC-OS). *European Heart Journal*.

[6] Mandala E, Lafara K, Kokkinovasilis D, Kalafatis I, Koukoulitsa V, Katodritou E (2024). Applied Cardio-Oncology in Hematological Malignancies: A Narrative Review. *Life (Basel, Switzerland)*.

[7] Herrmann J, McCullough KB, Habermann TM (2022). How I treat cardiovascular complications in patients with lymphoid malignancies. *Blood*.

[8] Wu L, Zhang Y, Wang G, Ren J (2024). Molecular Mechanisms and Therapeutic Targeting of Ferroptosis in Doxorubicin-Induced Cardiotoxicity. *JACC. Basic to Translational Science*.

[9] Barachini S, Buda G, Petrini I (2024). Cardiovascular Toxicity of Antineoplastic Treatments in Hematological Diseases: Focus on Molecular Mechanisms to Improve Therapeutic Management. *Journal of Clinical Medicine*.

[10] Herrmann J (2020). Vascular toxic effects of cancer therapies. *Nature Reviews. Cardiology*.

[11] Shi S, Chen Y, Luo Z, Nie G, Dai Y (2023). Role of oxidative stress and inflammation-related signaling pathways in doxorubicin-induced cardiomyopathy. *Cell Communication and Signaling: CCS*.

[12] Efentakis P, Varela A, Chavdoula E, Sigala F, Sanoudou D, Tenta R (2020). Levosimendan prevents doxorubicin-induced cardiotoxicity in time- and dose-dependent manner: implications for inotropy. *Cardiovascular Research*.

[13] Ma ZG, Kong CY, Wu HM, Song P, Zhang X, Yuan YP (2020). Toll-like receptor 5 deficiency diminishes doxorubicin-induced acute cardiotoxicity in mice. *Theranostics*.

[14] Alizadehasl A, Shahrami B, Rahbarghazi R, Yalameh Aliabadi A, Hosseini Jebelli SF, Afsari Zonooz Y (2024). Post-transplant cyclophosphamide-induced cardiotoxicity: A comprehensive review. *Journal of Cardiovascular and Thoracic Research*.

[15] Adhab AH, Altalbawy FMA, Mahdi MS, Baldaniya L, Omar TM, Ganesan S (2025). NADPH Oxidases in Cancer Therapy-Induced Cardiotoxicity: Mechanisms and Therapeutic Approaches. *Cardiovascular Toxicology*.

[16] Whitcomb LA, Cao X, Thomas D, Wiese C, Pessin AS, Zhang R (2024). Mitochondrial reactive oxygen species impact human fibroblast responses to protracted γ-ray exposures. *International Journal of Radiation Biology*.

[17] Yu QQ, Zhang H, Guo Y, Han B, Jiang P (2022). The Intestinal Redox System and Its Significance in Chemotherapy-Induced Intestinal Mucositis. *Oxidative Medicine and Cellular Longevity*.

[18] Aung LHH, Li R, Prabhakar BS, Li P (2017). Knockdown of Mtfp1 can minimize doxorubicin cardiotoxicity by inhibiting Dnm1l-mediated mitochondrial fission. *Journal of Cellular and Molecular Medicine*.

[19] Zeng H, Zou P, Chen Y, Zhang P, Shao L (2024). NOX4 aggravates doxorubicin-induced cardiomyocyte pyroptosis by increasing reactive oxygen species content and activating the NLRP3 inflammasome. *Cardiovascular Diagnosis and Therapy*.

[20] Wu HL, Gong Y, Ji P, Xie YF, Jiang YZ, Liu GY (2022). Targeting nucleotide metabolism: a promising approach to enhance cancer immunotherapy. *Journal of Hematology & Oncology*.

[21] Rezaei S, Mohammadzadeh-Vardin M, Amirshahrokhi K, Amani M (2025). The synergistic effect of 2-deoxy-D-glucose and cytarabine on mitochondria of stem-like cells derived from KG1-a. *Leukemia Research Reports*.

[22] Zhou N, Wei S, Sun T, Xie S, Liu J, Li W (2023). Recent progress in the role of endogenous metal ions in doxorubicin-induced cardiotoxicity. *Frontiers in Pharmacology*.

[23] Fang X, Wang H, Han D, Xie E, Yang X, Wei J (2019). Ferroptosis as a target for protection against cardiomyopathy. *Proceedings of the National Academy of Sciences of the United States of America*.

[24] Wang L, Liu Y, Du T, Yang H, Lei L, Guo M (2020). ATF3 promotes erastin-induced ferroptosis by suppressing system Xc. *Cell Death and Differentiation*.

[25] Miotto G, Rossetto M, Di Paolo ML, Orian L, Venerando R, Roveri A (2020). Insight into the mechanism of ferroptosis inhibition by ferrostatin-1. *Redox Biology*.

[26] Liu Y, Zeng L, Yang Y, Chen C, Wang D, Wang H (2020). Acyl-CoA thioesterase 1 prevents cardiomyocytes from Doxorubicin-induced ferroptosis via shaping the lipid composition. *Cell Death & Disease*.

[27] Wang X, Sun Q, Jiang Q, Jiang Y, Zhang Y, Cao J (2021). Cryptotanshinone Ameliorates Doxorubicin-Induced Cardiotoxicity by Targeting Akt-GSK-3β-mPTP Pathway In Vitro. *Molecules (Basel, Switzerland)*.

[28] Sun M, Zhang X, Tan B, Zhang Q, Zhao X, Dong D (2024). Potential role of endoplasmic reticulum stress in doxorubicin-induced cardiotoxicity-an update. *Frontiers in Pharmacology*.

[29] Zeng C, Duan F, Hu J, Luo B, Huang B, Lou X (2020). NLRP3 inflammasome-mediated pyroptosis contributes to the pathogenesis of non-ischemic dilated cardiomyopathy. *Redox Biology*.

[30] Nizami ZN, Aburawi HE, Semlali A, Muhammad K, Iratni R (2023). Oxidative Stress Inducers in Cancer Therapy: Preclinical and Clinical Evidence. *Antioxidants (Basel, Switzerland)*.

[31] Al-Kenany SA, Al-Shawi NN (2023). Protective effect of cafestol against doxorubicin-induced cardiotoxicity in rats by activating the Nrf2 pathway. *Frontiers in Pharmacology*.

[32] Baier MJ, Noack J, Seitz MT, Maier LS, Neef S (2021). Phosphorylation of RyR2 Ser-2814 by CaMKII mediates β1-adrenergic stress induced Ca2+ -leak from the sarcoplasmic reticulum. *FEBS Open Bio*.

[33] Yang Y, Wang Z, Wang N, Yang J, Yang L (2024). CaMKII Exacerbates Doxorubicin-Induced Cardiotoxicity by Promoting Ubiquitination Through USP10 Inhibition. *Cancer Medicine*.

[34] Uchida K, Scarborough EA, Prosser BL (2022). Cardiomyocyte Microtubules: Control of Mechanics, Transport, and Remodeling. *Annual Review of Physiology*.

[35] Nasilli G, de Waal TM, Marchal GA, Bertoli G, Veldkamp MW, Rothenberg E (2024). Decreasing microtubule detyrosination modulates Nav1.5 subcellular distribution and restores sodium current in mdx cardiomyocytes. *Cardiovascular Research*.

[36] Elmorsy EA, Saber S, Hamad RS, Abdel-Reheim MA, El-Kott AF, AlShehri MA (2024). Advances in understanding cisplatin-induced toxicity: Molecular mechanisms and protective strategies. *European Journal of Pharmaceutical Sciences: Official Journal of the European Federation for Pharmaceutical Sciences*.

[37] Nishikawa T, Miyahara E, Yamazaki I, Ikawa K, Nakagawa S, Kodama Y (2024). Effects of High-Dose Cyclophosphamide on Ultrastructural Changes and Gene Expression Profiles in the Cardiomyocytes of C57BL/6J Mice. *Diseases (Basel, Switzerland)*.

[38] Kreidieh F, McQuade J (2024). Novel insights into cardiovascular toxicity of cancer targeted and immune therapies: Beyond ischemia with non-obstructive coronary arteries (INOCA). *American Heart Journal Plus: Cardiology Research and Practice*.

[39] Terwoord JD, Beyer AM, Gutterman DD (2022). Endothelial dysfunction as a complication of anti-cancer therapy. *Pharmacology & Therapeutics*.

[40] Kattan LA, Abulola SM, Mohamed Ibrahim MI, Maayah ZH (2025). Anthracyclines-Induced Vascular Endothelial Dysfunction in Cancer Patients and Survivors Using Brachial Flow-Mediated Dilation (FMD) Tool: A Systematic Review and Meta-Analysis. *Cardiovascular Toxicology*.

[41] Zafar A, Drobni ZD, Lei M, Gongora CA, Quinaglia T, Lou UY (2022). The efficacy and safety of cardio-protective therapy in patients with 5-FU (Fluorouracil)-associated coronary vasospasm. *PloS One*.

[42] Clasen SC, Dinh PC, Hou L, Fung C, Sesso HD, Travis LB (2021). Cisplatin, environmental metals, and cardiovascular disease: an urgent need to understand underlying mechanisms. *Cardio-oncology (London, England)*.

[43] Kamath K, Smiyun G, Wilson L, Jordan MA (2014). Mechanisms of inhibition of endothelial cell migration by taxanes. *Cytoskeleton (Hoboken, N.J.)*.

[44] Assunção HC, Silva PMA, Bousbaa H, Cidade H (2025). Recent Advances in Microtubule Targeting Agents for Cancer Therapy. *Molecules (Basel, Switzerland)*.

[45] Belotti D, Vergani V, Drudis T, Borsotti P, Pitelli MR, Viale G (1996). The microtubule-affecting drug paclitaxel has antiangiogenic activity. *Clinical Cancer Research: an Official Journal of the American Association for Cancer Research*.

[46] Chounta S, Lemler S, Haddy N, Fresneau B, Mansouri I, Bentriou M (2023). The risk of valvular heart disease in the French Childhood Cancer Survivors’ Study: Contribution of dose-volume histogram parameters. *Radiotherapy and Oncology: Journal of the European Society for Therapeutic Radiology and Oncology*.

[47] Wilson J, Jun Hua C, Aziminia N, Manisty C (2025). Imaging of the Acute and Chronic Cardiovascular Complications of Radiation Therapy. *Circulation. Cardiovascular Imaging*.

[48] Uehara M, Bekki N, Shiga T (2024). Radiation-associated cardiovascular disease in patients with cancer: current insights from a cardio-oncologist. *Journal of Radiation Research*.

[49] Lee C, Hahn RT (2022). Valvular Heart Disease Associated With Radiation Therapy: A Contemporary Review. *Structural Heart: the Journal of the Heart Team*.

[50] Leerink JM, de Baat EC, Feijen EAM, Bellersen L, van Dalen EC, Grotenhuis HB (2020). Cardiac Disease in Childhood Cancer Survivors: Risk Prediction, Prevention, and Surveillance: JACC CardioOncology State-of-the-Art Review. *JACC. CardioOncology*.

[51] Bates JE, Shrestha S, Liu Q, Smith SA, Mulrooney DA, Leisenring W (2023). Cardiac Substructure Radiation Dose and Risk of Late Cardiac Disease in Survivors of Childhood Cancer: A Report From the Childhood Cancer Survivor Study. *Journal of Clinical Oncology: Official Journal of the American Society of Clinical Oncology*.

[52] Shrestha S, Bates JE, Liu Q, Smith SA, Oeffinger KC, Chow EJ (2021). Radiation therapy related cardiac disease risk in childhood cancer survivors: Updated dosimetry analysis from the Childhood Cancer Survivor Study. *Radiotherapy and Oncology: Journal of the European Society for Therapeutic Radiology and Oncology*.

[53] DeVine A, Landier W, Hudson MM, Constine LS, Bhatia S, Armenian SH (2025). The Children’s Oncology Group Long-Term Follow-Up Guidelines for Survivors of Childhood, Adolescent, and Young Adult Cancers: A Review. *JAMA Oncology*.

[54] Ibáñez B, Melero A, Montoro A, San Onofre N, Soriano JM (2024). Molecular Insights into Radiation Effects and Protective Mechanisms: A Focus on Cellular Damage and Radioprotectors. *Current Issues in Molecular Biology*.

[55] Che M, Duan Y, Yin R (2024). A bibliometric analysis of cardiotoxicity in cancer radiotherapy. *Frontiers in Oncology*.

[56] Livingston K, Schlaak RA, Puckett LL, Bergom C (2020). The Role of Mitochondrial Dysfunction in Radiation-Induced Heart Disease: From Bench to Bedside. *Frontiers in Cardiovascular Medicine*.

[57] Zheng M, Liu Z, He Y (2024). Radiation-induced fibrosis: Mechanisms and therapeutic strategies from an immune microenvironment perspective. *Immunology*.

[58] Dreyfuss AD, Velalopoulou A, Avgousti H, Bell BI, Verginadis II (2022). Preclinical models of radiation-induced cardiac toxicity: Potential mechanisms and biomarkers. *Frontiers in Oncology*.

[59] Wijerathne H, Langston JC, Yang Q, Sun S, Miyamoto C, Kilpatrick LE (2021). Mechanisms of radiation-induced endothelium damage: Emerging models and technologies. *Radiotherapy and Oncology: Journal of the European Society for Therapeutic Radiology and Oncology*.

[60] Wasim S, Park J, Nam S, Kim J (2023). Review of Current Treatment Intensification Strategies for Prostate Cancer Patients. *Cancers*.

[61] Bates JE, Rancati T, Keshavarz H, Gagliardi G, Aznar MC, Howell RM (2024). Cardiac Disease in Childhood Cancer Survivors Treated With Radiation Therapy: A PENTEC Comprehensive Review. *International Journal of Radiation Oncology, Biology, Physics*.

[62] Mahadeo KM, Khazal SJ, Abdel-Azim H, Fitzgerald JC, Taraseviciute A, Bollard CM (2019). Management guidelines for paediatric patients receiving chimeric antigen receptor T cell therapy. *Nature Reviews. Clinical Oncology*.

[63] Ferreri CJ, Bhutani M (2024). Mechanisms and management of CAR T toxicity. *Frontiers in Oncology*.

[64] Demosthenous C, Evangelidis P, Gatsis A, Mitroulis I, Vakalopoulou S, Vardi A (2025). Endothelial Injury Following CAR-T Cell Immunotherapy for Hematological Malignancies. *Cancers*.

[65] Zandaki D, Selukar S, Bi Y, Li Y, Zinsky M, Bonifant CL (2025). EASIX and m-EASIX predict CRS and ICANS in pediatric and AYA patients after CD19-CAR T-cell therapy. *Blood Advances*.

[66] Lee DH, Jain M, Lazaryan A, Locke FL, Jeong D, Alomar M (2022). Case of Myocarditis After Chimeric Antigen Receptor T Cells With Intracardiac Lymphoma. *JACC. Case Reports*.

[67] Morris EC, Neelapu SS, Giavridis T, Sadelain M (2022). Cytokine release syndrome and associated neurotoxicity in cancer immunotherapy. *Nature Reviews. Immunology*.

[68] Dawn B, Xuan YT, Guo Y, Rezazadeh A, Stein AB, Hunt G (2004). IL-6 plays an obligatory role in late preconditioning via JAK-STAT signaling and upregulation of iNOS and COX-2. *Cardiovascular Research*.

[69] Hay KA, Hanafi LA, Li D, Gust J, Liles WC, Wurfel MM (2017). Kinetics and biomarkers of severe cytokine release syndrome after CD19 chimeric antigen receptor-modified T-cell therapy. *Blood*.

[70] Munir M, Sayed A, Addison D, Epperla N (2024). Cardiovascular toxicities associated with novel cellular immune therapies. *Blood Advances*.

[71] Maleki S, Esmaeili Z, Seighali N, Shafiee A, Namin SM, Zavareh MAT (2024). Cardiac adverse events after Chimeric Antigen Receptor (CAR) T cell therapies: an updated systematic review and meta-analysis. *Cardio-oncology (London, England)*.

[72] Lazzerini PE, Abbate A, Boutjdir M, Capecchi PL (2023). Fir(e)ing the Rhythm: Inflammatory Cytokines and Cardiac Arrhythmias. *JACC. Basic to Translational Science*.

[73] Montalvo MJ, Bandey IN, Rezvan A, Wu KL, Saeedi A, Kulkarni R (2024). Decoding the mechanisms of chimeric antigen receptor (CAR) T cell-mediated killing of tumors: insights from granzyme and Fas inhibition. *Cell Death & Disease*.

[74] Klampatsa A, Leibowitz MS, Sun J, Liousia M, Arguiri E, Albelda SM (2020). Analysis and Augmentation of the Immunologic Bystander Effects of CAR T Cell Therapy in a Syngeneic Mouse Cancer Model. *Molecular Therapy Oncolytics*.

[75] Ehrlich P, Klingel K, Ohlmann-Knafo S, Hüttinger S, Sood N, Pickuth D (2021). Biopsy-proven lymphocytic myocarditis following first mRNA COVID-19 vaccination in a 40-year-old male: case report. *Clinical Research in Cardiology: Official Journal of the German Cardiac Society*.

[76] Linette GP, Stadtmauer EA, Maus MV, Rapoport AP, Levine BL, Emery L (2013). Cardiovascular toxicity and titin cross-reactivity of affinity-enhanced T cells in myeloma and melanoma. *Blood*.

[77] Giavridis T, van der Stegen SJC, Eyquem J, Hamieh M, Piersigilli A, Sadelain M (2018). CAR T cell-induced cytokine release syndrome is mediated by macrophages and abated by IL-1 blockade. *Nature Medicine*.

[78] Kerr WG, Chisholm JD (2019). The Next Generation of Immunotherapy for Cancer: Small Molecules Could Make Big Waves. *Journal of Immunology (Baltimore, Md.: 1950)*.

[79] Johnson DB, Balko JM, Compton ML, Chalkias S, Gorham J, Xu Y (2016). Fulminant Myocarditis with Combination Immune Checkpoint Blockade. *The New England Journal of Medicine*.

[80] Jain V, Bahia J, Mohebtash M, Barac A (2017). Cardiovascular Complications Associated With Novel Cancer Immunotherapies. *Current Treatment Options in Cardiovascular Medicine*.

[81] Heinzerling L, Ott PA, Hodi FS, Husain AN, Tajmir-Riahi A, Tawbi H (2016). Cardiotoxicity associated with CTLA4 and PD1 blocking immunotherapy. *Journal for Immunotherapy of Cancer*.

[82] Behling J, Kaes J, Münzel T, Grabbe S, Loquai C (2017). New-onset third-degree atrioventricular block because of autoimmune-induced myositis under treatment with anti-programmed cell death-1 (nivolumab) for metastatic melanoma. *Melanoma Research*.

[83] Läubli H, Balmelli C, Bossard M, Pfister O, Glatz K, Zippelius A (2015). Acute heart failure due to autoimmune myocarditis under pembrolizumab treatment for metastatic melanoma. *Journal for Immunotherapy of Cancer*.

[84] Semper H, Muehlberg F, Schulz-Menger J, Allewelt M, Grohé C (2016). Drug-induced myocarditis after nivolumab treatment in a patient with PDL1- negative squamous cell carcinoma of the lung. *Lung Cancer (Amsterdam, Netherlands)*.

[85] Mahmood SS, Fradley MG, Cohen JV, Nohria A, Reynolds KL, Heinzerling LM (2018). Myocarditis in Patients Treated With Immune Checkpoint Inhibitors. *Journal of the American College of Cardiology*.

[86] Vergara A, De Felice M, Cesaro A, Gragnano F, Pariggiano I, Golia E (2024). Immune-Checkpoint Inhibitor-Related Myocarditis: Where We Are and Where We Will Go. *Angiology*.

[87] Francisco LM, Sage PT, Sharpe AH (2010). The PD-1 pathway in tolerance and autoimmunity. *Immunological Reviews*.

[88] Vasbinder A, Hoeger CW, Catalan T, Anderson E, Chu C, Kotzin M (2023). Cardiovascular Events After Hematopoietic Stem Cell Transplant: Incidence and Risk Factors. *JACC. CardioOncology*.

[89] Huang YV, Waliany S, Lee D, Galdos FX, Witteles RM, Neal JW (2022). The Role of Single-Cell Profiling and Deep Immunophenotyping in Understanding Immune Therapy Cardiotoxicity. *JACC. CardioOncology*.

[90] Jiménez-Alejandre R, Ruiz-Fernández I, Martín P (2022). Pathophysiology of Immune Checkpoint Inhibitor-Induced Myocarditis. *Cancers*.

[91] Moslehi J, Lichtman AH, Sharpe AH, Galluzzi L, Kitsis RN (2021). Immune checkpoint inhibitor-associated myocarditis: manifestations and mechanisms. *The Journal of Clinical Investigation*.

[92] Jo W, Won T, Daoud A, Čiháková D (2024). Immune checkpoint inhibitors associated cardiovascular immune-related adverse events. *Frontiers in Immunology*.

[93] Čiháková D (2024). T Cells and Macrophages Drive Pathogenesis of Immune Checkpoint Inhibitor Myocarditis. *Circulation*.

[94] Kroll MH, Rojas-Hernandez C, Yee C (2022). Hematologic complications of immune checkpoint inhibitors. *Blood*.

[95] Li X, Peng W, Wu J, Yeung SCJ, Yang R (2023). Advances in immune checkpoint inhibitors induced-cardiotoxicity. *Frontiers in Immunology*.

[96] Wang WX, Song ZZ, Zhang YP (2020). Cardiovascular toxicities associated with immune checkpoint inhibitors. *Zhonghua Zhong Liu Za Zhi*.

[97] Gao Y, Zhang H, Qiu Y, Bian X, Wang X, Li Y (2024). Early identification of severe immune checkpoint inhibitor associated myocarditis: From an electrocardiographic perspective. *Cancer Medicine*.

[98] Hayek SS, Zaha VG, Bogle C, Deswal A, Langston A, Rotz S (2024). Cardiovascular Management of Patients Undergoing Hematopoietic Stem Cell Transplantation: From Pretransplantation to Survivorship: A Scientific Statement From the American Heart Association. *Circulation*.

[99] Hundal J, Curley T, Hamilton BK (2024). Cardiovascular Considerations in Patients Undergoing Hematopoietic Cell Transplantation. *Current Treatment Options in Oncology*.

[100] Agarwal N, Rotz S, Hanna R (2023). Medical emergencies in pediatric blood & marrow transplant and cellular therapies. *Frontiers in Pediatrics*.

[101] Moreno-Castaño AB, Salas MQ, Palomo M, Martinez-Sanchez J, Rovira M, Fernández-Avilés F (2022). Early vascular endothelial complications after hematopoietic cell transplantation: Role of the endotheliopathy in biomarkers and target therapies development. *Frontiers in Immunology*.

[102] Evangelidis P, Evangelidis N, Kalmoukos P, Kourti M, Tragiannidis A, Gavriilaki E (2024). Genetic Susceptibility in Endothelial Injury Syndromes after Hematopoietic Cell Transplantation and Other Cellular Therapies: Climbing a Steep Hill. *Current Issues in Molecular Biology*.

[103] Hauwanga WN, McBenedict B, Amadi ES, Dohadwala TK, Johnny C, Asaju F (2024). A Systematic Review of the Cardiotoxic Effects of Targeted Therapies in Oncology. *Cureus*.

[104] Ata F, Benkhadra M, Ghasoub R, Fernyhough LJ, Omar NE, Nashwan AJ (2023). Tyrosine Kinase Inhibitors in pediatric chronic myeloid leukemia: a focused review of clinical trials. *Frontiers in Oncology*.

[105] le Noble FAC, Mourad JJ, Levy BI, Struijker-Boudier HAJ (2023). VEGF (Vascular Endothelial Growth Factor) Inhibition and Hypertension: Does Microvascular Rarefaction Play a Role?. *Hypertension (Dallas, Tex.: 1979)*.

[106] Kuang H, Yan Q, Li Z, Lin A, Li K, Zhang J (2024). Comprehensive analysis of VEGF/VEGFR inhibitor-induced immune-mediated hypertension: integrating pharmacovigilance, clinical data, and preclinical models. *Frontiers in Immunology*.

[107] Pollard JA, Alonzo TA, Gerbing R, Brown P, Fox E, Choi J (2022). Sorafenib in Combination With Standard Chemotherapy for Children With High Allelic Ratio FLT3/ITD+ Acute Myeloid Leukemia: A Report From the Children’s Oncology Group Protocol AAML1031. *Journal of Clinical Oncology: Official Journal of the American Society of Clinical Oncology*.

[108] Mertens L, Singh G, Armenian S, Chen MH, Dorfman AL, Garg R (2023). Multimodality Imaging for Cardiac Surveillance of Cancer Treatment in Children: Recommendations From the American Society of Echocardiography. *Journal of the American Society of Echocardiography: Official Publication of the American Society of Echocardiography*.

[109] Ryan TD, Bates JE, Kinahan KE, Leger KJ, Mulrooney DA, Narayan HK (2025). Cardiovascular Toxicity in Patients Treated for Childhood Cancer: A Scientific Statement From the American Heart Association. *Circulation*.

[110] Thomas JD, Edvardsen T, Abraham T, Appadurai V, Badano L, Banchs J (2025). Clinical Applications of Strain Echocardiography: A Clinical Consensus Statement From the American Society of Echocardiography Developed in Collaboration With the European Association of Cardiovascular Imaging of the European Society of Cardiology. *Journal of the American Society of Echocardiography: Official Publication of the American Society of Echocardiography*.

